# Dyspepsia

**Published:** 1871-01

**Authors:** 


### DYSPEPSIA

Is a weak stomach, made weak by overwork, and, like a man made weak by overwork, it needs rest, needs repose; but as we cannot live without eating, the necessity must be met by giving the stomach as little work to do as possible, and that work should be easy, just as we ourselves, in the weakness of recovery from disease, invite our strength back by doing but little work, and that which can be easily done.

A man recovering from brain-fever, which has left that important organ in a state of distressing debility, would destroy himself if he were to attempt the most abstruse questions in astronomy, mathematics, or prophecy.

The cure of most cases of dyspepsia becomes extremely simple and very certain, if these few first principles are judiciously applied in any given case, to wit: give the stomach but little to do, let that little be of a kind which is easily done, and let both be so arranged that the stomach may do its work easily and soon, and have abundant time for rest. The work of the stomach is called "digestion," and means the process of preparing the food for yielding its nutrient portions to the system, to give it warmth, growth, and strength, as explained more fully in the book entitled

"HEALTH BY GOOD LIVING."

The food to be eaten to cure dyspepsia — medicine can never cure it — must not only be small in quantity and easily digested, but it must be nutritious, and have a good deal of waste, — must not be concentrated food.

Fresh meats, beef, mutton, poultry, fish, answer all these conditions admirably, the two first named being preferable. Physiologists assert that meat, being so near alike the flesh of the body already, requires, under all the circumstances, less work for the stomach to convert it into our own flesh and blood than any other food in dyspepsia. Take but little of it at a time; a faithful servant, weak from recent disease, can do a little work, and can do it well; if a heavy task is imposed, in the effort to do it all well, none of it is well done. A weak stomach may perhaps be able to work up a large amount of food, but it is imperfectly done; it will not be "accepted" by watchful nature; hence the universal complaint of dyspeptics, "I eat a great deal, but it seems to do me no good."

A person left excessively weak by recent disease, but beginning to recover, walks a short distance, walks a very few minutes, and rests a long time; common sense teaches that; so a weak, which is a dyspeptic, stomach, working, exercising a little and resting a long time, gains power for more work, harder work ; hence the need of giving but little food at a time and a great deal of rest afterwards. Porter-house steak, roast beef, and mutton require three hours for digestion, and to be passed out of the stomach, when eaten with a relish. The stomach, having to work three hours, ought to have at least two hours' rest ; hence a dyspeptic should not eat oftener than once in five hours during daylight, in ordinary circumstances. The food eaten in dyspepsia should have a good deal of waste matter, in order, among other things, to have waste matter enough to form material to cause a daily action of the system ; about two thirds of roast beef is waste matter. Concentrated food, such as rice, beans, and the like, which are almost all nutriment, "bind" the system, make it "feverish," as every farmer knows that oats will do, if made the exclusive food of horses. The remedy is roughness, fodder, hay and straw, which have great bulk, but comparatively little nourishment. The main food, then, for the dyspeptic is meat, to which should be added the crust of loaf bread, because light, or bread made of the whole product of the grain of wheat. Bread is added to meat, because it alone has all the elements of nutrition meat has not.

As a general thing, dyspeptics should not drink anything at meals, because there is a liquid in the stomach which dissolves the food, — in a sense melts it. If cold water is drank, it cools this stomach liquid, and it loses its power of melting the food, so to speak ; as the cooler the water is, the less is it able to melt the ice in it. Of course every physiologist knows that this comparison is not critically true ; but it conveys the essential practical idea to the minds of the masses. If a man drinks a certain amount of pure brandy, his head will begin to swim, he will be drunk in a few minutes ; if that brandy is diluted with water, it will have less effect. Some liquids will dissolve solid iron ; a handful of small nails will be "eaten up," will be dissolved in cider, sooner in vinegar, because the latter has less water than cider; so also if the liquid in the stomach is diluted with any other liquid, it is just that much powerless to dissolve the food eaten. Hence, as a general rule, the less of any fluid taken by the dyspeptic while eating, the better; but in an hour or two after eating, after the food is mainly dissolved, as much liquid may be used as the thirst requires. Drink nothing without thirst, and then cold water.

